# Supplementary material for: Comparing Bayesian-Based Reconstruction Strategies in Topology-Based Pathway Enrichment Analysis
Source: Biomolecules. 2022 Jun 28;12(7):906. doi: 10.3390/biom12070906 (PMC9313337; doi:10.3390/biom12070906)
Supplement: Supplementary file 1 [file biomolecules-12-00906-s001.zip › S0_Supplementary_Algorithm.pdf]

Table S1 BPA Reconstruction

Input: KEGG pathway with node list N and edge list E;

seeds = {};
Initialization cyclic graph G with nodes N and edges E;
Run Tarjan's algorithm to detect all cyclegroups $(C_1, C_2, \cdots, C_n)$;
Remove all edges connecting nodes in the same cyclegroup;
for C_i in $(C_1, C_2, \cdots, C_n)$ **do**
    Randomly number each node inside C_i from small to large;
    Add an edge from each lower number node to each higher number node;
    For each parent A of a member of the cycle group that is not itself in the cyclegroup, add an edge from A to each member of the cyclegroup;
end for

Output: Reconstructed pathway directed acyclic graph.

Table S2 PROPS Reconstruction

Input: KEGG pathway with node list N and edge list E;

seeds = {};
Initialization empty graph G with nodes N from the input pathway;
while $E \neq \emptyset$ **do**
    Randomly select e_i from E;
    Add e_i to the graph G;
    if cyclic(G) **then**
        Remove e_i from the graph G;
    end if
    Remove e_i from the edge list E;
end while

Output: Reconstructed pathway directed acyclic graph.

Table S3 Clipper Reconstruction

Input: KEGG pathway with node list N and edge list E, healthy RNA-seq data H;

for n_i in N **do**
    Extract parent node set N_p for n_i according to the edge list;
    Extract corresponding expression data from healthy samples (H_{N_p} and H_{n_i}) for parent genes N_p and children gene n_i;

4: Fit linear regression model for healthy samples lm($H_{ni}\sim H_{Np}$);

5: **for** n_j in N_p **do**

6: Assign p-value p_{nj} to the edge from n_j to n_i;

7: **end for** 8: **end for**

9: Sort the list of edges in ascending p-value order;

10: **Initialization** empty graph G with nodes N from the input pathway;

11: **while** E/= ∅ **do**

12: Add edge e_i to the graph G in ascending p-value order;

13: **if** cyclic(G) **then**

14: Remove e_i from the graph G;

15: **end if**

16: Remove e_i from the edge list E;

17: **end while**

Output: Reconstructed pathway directed acyclic graph.

Table S4 BNrich Reconstruction

Input: KEGG pathway with node list N and edge list E, healthy and disease RNA-seq data H and D;

Stage 1: Eliminate edges through biological approaches;

1: **for** e_i in E **do**

2: **if** (

3: e_i connects two nodes with same label;

4: e_i originates from nucleus resulting in cycle formation;

5: e_i is opposite of the signaling flux direction and would lead to cycle ;

6:) {

7: Remove e_i from the edge list;

8: }

9: **end for**

Stage 2: Simplify structure by LASSO

10: **for** n_i in N **do**

11: Extract parent node set N_p for n_i according to the edge list;

12: Extract corresponding expression data from healthy (H_{Np} and H_{ni}) and disease (D_{Np} and D_{ni}) samples for parent genes N_p and children gene n_i;

13: Fit regression model for healthy and disease sample separately LASSO($H_{ni}\sim H_{Np}$) and LASSO($D_{ni}\sim D_{Np}$);

for n_j in N_p **do**
if $\beta_{n_j} = 0$ in both models **then**
Remove the edge from n_j to n_i;
end if
end for
end for
Output: Simplified pathway directed acyclic graph.

Table S5 Ensemble Reconstruction

Input: KEGG pathway with node list N and edge list E;
seeds = {};
Initialization cyclic graph G with nodes N and edges E;
Run Tarjan's algorithm to detect all strongly connected components (SCCs) ($SCC_1, SCC_2, \cdots, SCC_n$);
for metric in (TrueSkill, Social Agony) **do**
for strategy in (Forward, Backward, Greedy) **do**
for SCC_i in ($SCC_1, SCC_2, \cdots, SCC_n$) **do**
if metric == TrueSkill **then**
For each edge (u,v):
if v has a higher skill level than u (expected) **then**
Update the skill level μ and σ of u by a small amount;
else
Update the skill level μ and σ of u to a large extent;
end if
The ranking score of node u is defined as $\mu_u - 3\sigma_u$;
else if metric == Social Agony **then**
Update ranking scores of each node by minimizing the total agony:
$A(G) = min_r(\sum_{(u,v)\in E} \max(r(u) - r(v) + 1, 0))$
end if
if strategy == Forward **then**
Select the node v which has the highest ranking score in SCC_i;
Remove its all out edges in SCC_i;
else if strategy == Backward **then**
Select the node v which has the lowest ranking score in SCC_i;
Remove its all in edges in SCC_i;
else if strategy == Greedy **then**
Select the edge with the highest difference in ranking score between the starting and ending nodes to remove;

end if
end for
end for
end for
For each edge (u,v), add the number of removals under the 6 conditions;
Remove edge in the order of highest to lowest voting scores until the graph becomes acyclic;

Output: Reconstructed pathway directed acyclic graph.
