# Supplementary material for: Comparing Bayesian-Based Reconstruction Strategies in Topology-Based Pathway Enrichment Analysis
Source: Biomolecules. 2022 Jun 28;12(7):906. doi: 10.3390/biom12070906 (PMC9313337; doi:10.3390/biom12070906)
Supplement: Supplementary file 1 [file biomolecules-12-00906-s001.zip › S2_Supplementary File 2 - Top_pathways.pdf]

Supplementary Table S1. The top ten pathways enriched by any of the five BN reconstruction methods in HNSC dataset.

Pathway Name	Rank in BNrich	Rank in PROPS	Rank in Clipper	Rank in BPA	Rank in Ensemble
**Adherens junction**	60	65	69	4	65
**AGE-RAGE signaling pathway in diabetic complications**	15	7	8	31	5
**Alcoholism**	14	9	9	25	6
**Axon guidance**	56	39	65	7	42
**Basal cell carcinoma**	140	11	43	35	9
**Central carbon metabolism in cancer**	6	34	12	19	11
**Chemical carcinogenesis - DNA adducts**	5	3	3	2	7
**Chemokine signaling pathway**	4	NA	NA	NA	NA
**Circadian entrainment**	25	27	10	27	80
**ECM-receptor interaction**	2	1	1	3	2
**Endocrine resistance**	16	8	4	5	4
**Focal adhesion**	10	NA	NA	NA	NA
**JAK-STAT signaling pathway**	1	NA	NA	NA	NA
**MAPK signaling pathway**	8	41	50	NA	31
**NF-kappa B signaling pathway**	42	12	57	8	37
**p53 signaling pathway**	7	51	35	21	25
**Pancreatic cancer**	34	5	5	9	24
**PI3K-Akt signaling pathway**	3	2	2	1	1
**Prostate cancer**	18	10	13	22	10
**Ras signaling pathway**	9	17	53	34	19
**Regulation of actin cytoskeleton**	12	16	11	6	8
**Small cell lung cancer**	145	4	7	15	3
**Type II diabetes mellitus**	98	99	40	10	101
**Wnt signaling pathway**	11	6	6	13	12

*NA: Pathways failed to be reconstructed into Bayesian network.

Supplementary Table S2. The top ten pathways enriched by any of the five BN reconstruction methods in KIRC dataset.

Pathway Name	Rank in BNrich	Rank in PROPS	Rank in Clipper	Rank in BPA	Rank in Ensemble
**Adherens junction**	19	3	23	19	12
**Adipocytokine signaling pathway**	10	93	19	54	26
**Cell adhesion molecules**	33	10	28	16	40
**Chemokine signaling pathway**	85	6	7	11	6
**Circadian entrainment**	49	15	25	4	10
**ECM-receptor interaction**	2	8	6	13	2
**EGFR tyrosine kinase inhibitor resistance**	7	45	9	5	17
**Insulin resistance**	29	5	16	30	16
**JAK-STAT signaling pathway**	18	NA	8	3	5
**Longevity regulating pathway**	6	9	22	9	30
**Long-term depression**	8	28	13	21	24
**MAPK signaling pathway**	3	27	49	39	51
**Morphine addiction**	14	1	12	2	13
**NF-kappa B signaling pathway**	56	7	68	90	124
**Pathways in cancer**	16	14	2	6	9
**PI3K-Akt signaling pathway**	1	26	3	14	3
**Prostate cancer**	9	17	11	10	8
**Rap1 signaling pathway**	5	13	5	8	7
**Ras signaling pathway**	4	11	4	12	4
**Regulation of actin cytoskeleton**	21	4	31	24	15
**Retrograde endocannabinoid signaling**	113	2	1	1	1
**Small cell lung cancer**	11	12	10	7	11

*NA: Pathways failed to be reconstructed into Bayesian network.

Supplementary Table S3. The top ten pathways enriched by any of the five BN reconstruction methods in LUAD dataset.

Pathway Name	Rank in BNrich	Rank in PROPS	Rank in Clipper	Rank in BPA	Rank in Ensemble
**AGE-RAGE signaling pathway in diabetic complications**	71	10	56	70	64
**AMPK signaling pathway**	12	48	30	46	10
**Apoptosis**	31	94	31	7	30
**Bladder cancer**	58	6	2	4	9
**Chagas disease**	25	7	33	2	16
**Chemokine signaling pathway**	29	13	5	25	7
**Cytokine-cytokine receptor interaction**	8	26	18	18	8
**Endocrine resistance**	14	41	4	6	3
**Fc epsilon RI signaling pathway**	3	1	45	19	17
**Focal adhesion**	9	NA	23	NA	4
**GABAergic synapse**	42	31	50	1	71
**Glioma**	18	24	35	16	5
**Herpes simplex virus 1 infection**	2	100	149	112	76
**HIF-1 signaling pathway**	1	15	20	10	26
**Human T-cell leukemia virus 1 infection**	115	9	22	52	31
**Huntington disease**	54	56	9	20	32
**JAK-STAT signaling pathway**	5	NA	21	21	29
**Leukocyte transendothelial migration**	39	66	8	50	1
**Longevity regulating pathway**	32	25	6	24	24
**Long-term depression**	4	39	7	3	33
**Melanoma**	37	20	1	37	21
**Morphine addiction**	68	8	46	22	12
**Natural killer cell mediated cytotoxicity**	33	3	26	26	70
**PI3K-Akt signaling pathway**	28	17	10	34	23
**Platinum drug resistance**	10	29	16	15	25
**Prion disease**	6	19	11	35	6
**Rap1 signaling pathway**	7	32	13	23	27

**Ras signaling pathway**	30	5	60	5	55
**Retrograde endocannabinoid signaling**	97	38	15	8	11
**Type II diabetes mellitus**	56	43	3	9	14
**Vascular smooth muscle contraction**	15	2	17	14	2
**Wnt signaling pathway**	24	4	24	27	15

*NA: Pathways failed to be reconstructed into Bayesian network.

Supplementary Table S4. The top ten pathways enriched by any of the five BN reconstruction methods in THCA dataset.

Pathway Name	Rank in BNrich	Rank in PROPS	Rank in Clipper	Rank in BPA	Rank in Ensemble
**AGE-RAGE signaling pathway in diabetic complications**	11	2	7	17	8
**Axon guidance**	7	3	16	56	18
**cAMP signaling pathway**	5	17	13	9	7
**Cell cycle**	49	6	32	57	42
**Chagas disease**	126	9	152	116	103
**Chemical carcinogenesis - DNA adducts**	27	1	24	27	27
**Chemokine signaling pathway**	19	39	18	6	6
**Complement and coagulation cascades**	23	16	6	14	10
**Focal adhesion**	34	NA	2	3	3
**Insulin resistance**	151	7	150	42	133
**JAK-STAT signaling pathway**	4	NA	4	4	5
**MAPK signaling pathway**	9	52	12	5	17
**Pathways in cancer**	24	10	8	12	4
**Phospholipase D signaling pathway**	45	15	9	8	11
**PI3K-Akt signaling pathway**	10	5	3	2	2
**PPAR signaling pathway**	3	4	1	1	1
**Proteoglycans in cancer**	8	23	15	10	14
**Rap1 signaling pathway**	2	11	20	13	13
**Ras signaling pathway**	1	12	5	7	9
**Regulation of actin cytoskeleton**	6	25	10	16	12
**Wnt signaling pathway**	31	8	23	26	36

*NA: Pathways failed to be reconstructed into Bayesian network.
